# Supplementary material for: Sustainable and Selective Extraction of Lipids and Bioactive Compounds from Microalgae
Source: Molecules. 2019 Nov 28;24(23):4347. doi: 10.3390/molecules24234347 (PMC6930523; doi:10.3390/molecules24234347)
Supplement: Supplementary file 1 [file molecules-24-04347-s001.pdf]

molecules

Sustainable and Selective Extraction of Lipids and Bioactive Compounds from Microalgae

Ilaria Santoro [1,*] Monica Nardi [2,*] Cinzia Benincasa [3] Paola Costanzo [2] Girolamo Giordano [1] Antonio Procopio [2] and Giovanni Sindona [4]

[1] *Dipartimento di* Ingegneria per l'Ambiente e il Territorio e Ingegneria Chimica, Università della Calabria, Cubo 45A, I-87036 Rende, Italy; girolamo.giordano@unical.it
[2] Dipartimento di Scienze della Salute, Università Magna Græcia, Viale Europa, I-88100 Germaneto (CZ), Italy; pcostanzo@unicz.it (P.C.); procopio@unicz.it (A.P.)
[3] CREA Research Centre for Olive, Citrus and Tree Fruit, C.da Li Rocchi, I-87036 Rende, Italy; cinzia.benincasa@crea.gov.it
[4] Dipartimento di Chimica e Tecnologie Chimiche, Università della Calabria, Cubo 12C, I-87036 Rende, Italy; giovanni.sindona@unical.it
* Correspondence: ilaria.santoro@unical.it (I.S.); monica.nardi@unicz.it (M.N.); Tel.: +39 0961 3694116 (M.N.)

Electronic Supplementary Material

Table of Contents

Experimental Section
**S1.** GC/MS chromatogram of algal oil sample.
Mass spectrum of miristate methyl ester
Mass spectrum of palmitate methyl ester
Mass spectrum of eicosapentaenoate methyl ester
Mass spectrum of docohexanoate methyl ester
**Table S1:** % FAMEs extracted

**S2.** Results of quantitative analysis of all fatty acids methyl esters.
Method Validation LC-MS
**Table S2**. List of the deprotonated molecules identified in full scan MS spectra of algal oil sample, fragment ions and precursor ions identified in MS/MS spectra.
**Table S3.** Molecular composition of adduct with negative molecular ion between m/z 455 and m/z 656.
**S3**.Negative ESI full scan mass spectrum of algal oil sample.
**S4**. Negative ESI full scan mass spectrum of standard solution of DHA, Stearic acid and butyric acid.
**S5**. Negative ESI full scan mass spectrum of standard solution of oleic alcohol and palmitic acid.

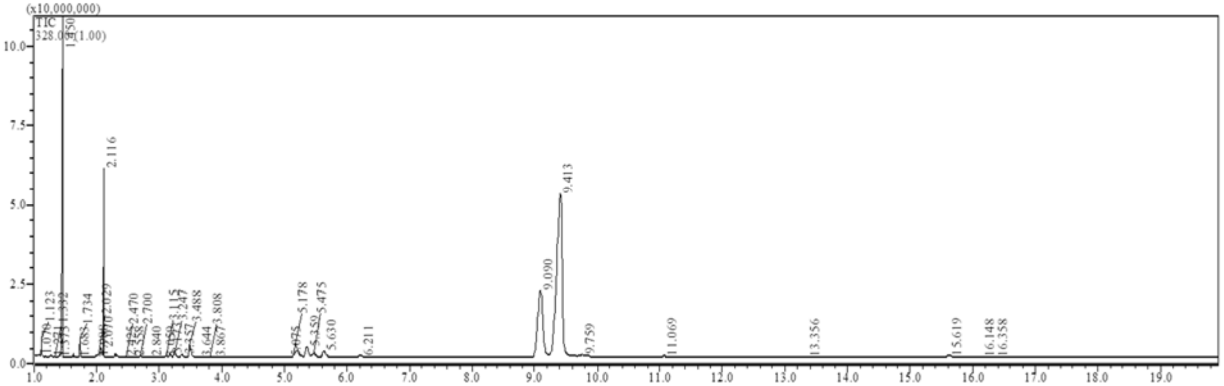

S1. GC/MS chromatogram of algal oil sample.

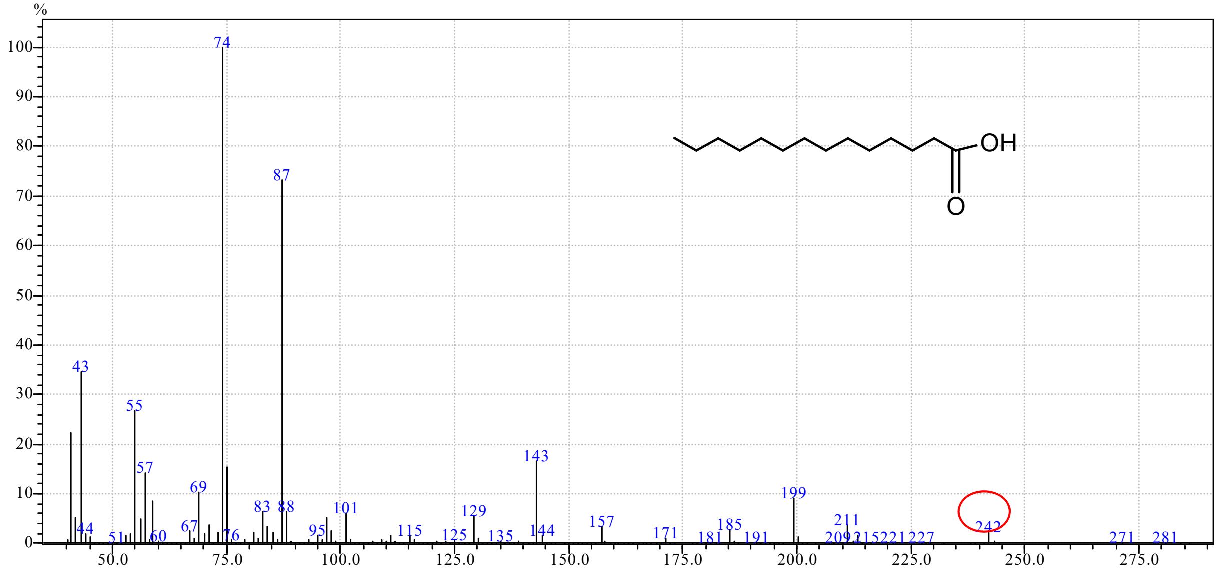

Mass spectrum of miristate methyl ester.

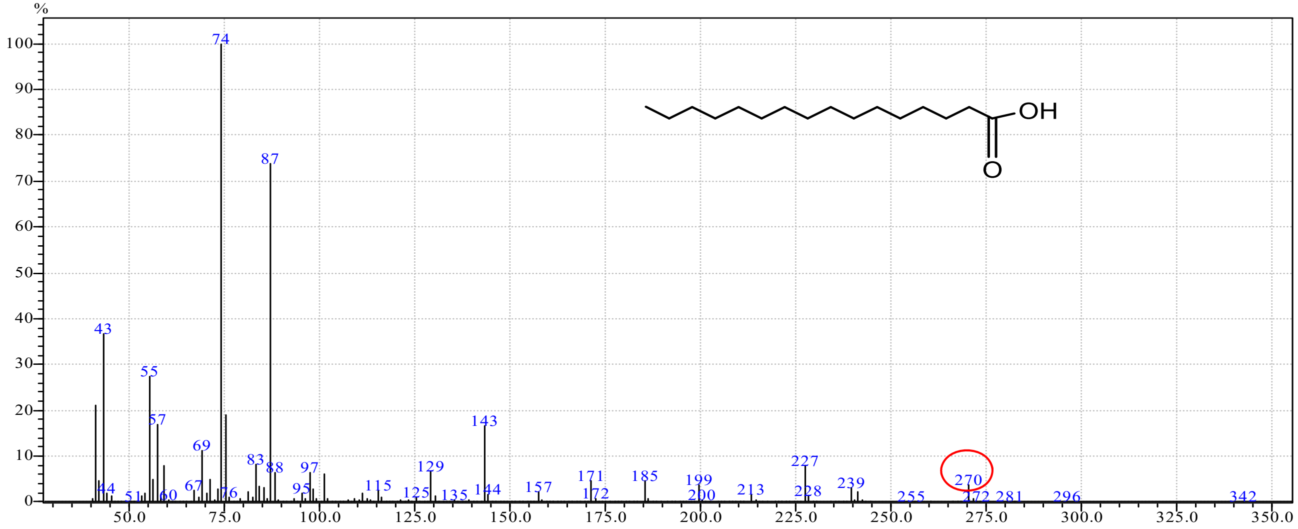

Mass spectrum of palmitate methyl ester

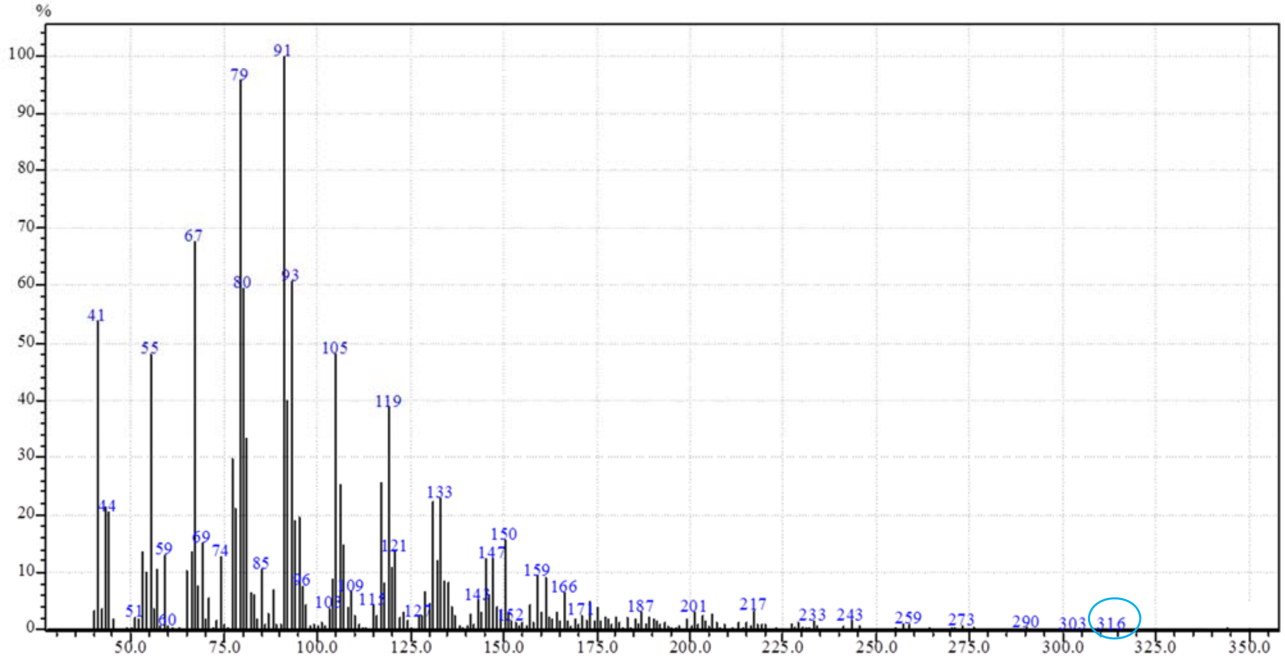

Mass spectrum of eicosapentaenoate methyl ester.

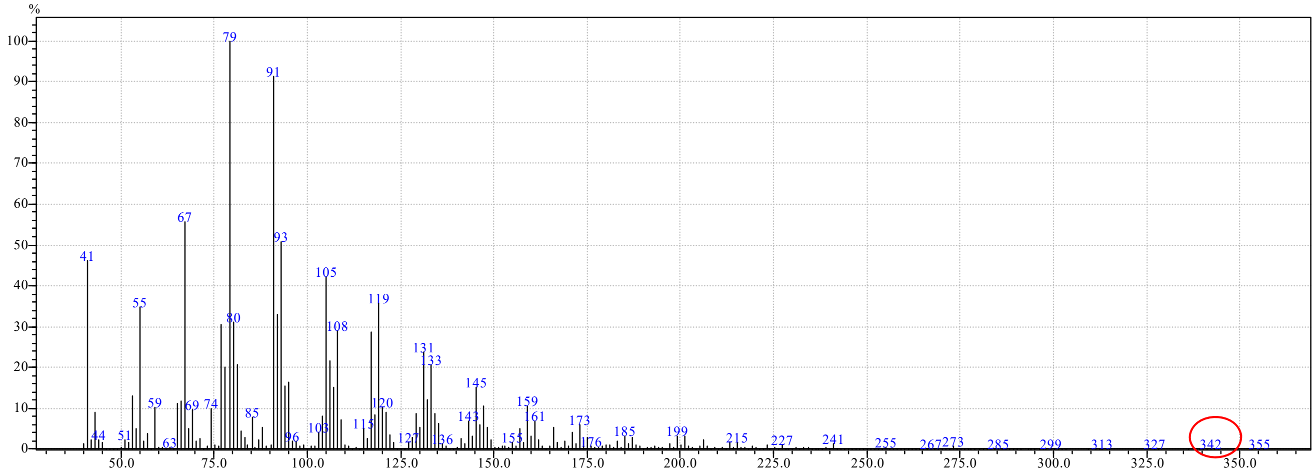

Mass spectrum of docohexanoate methyl ester

Table 1. % FAMEs extracted.

EtOH	14:0	15:0	16:0	17:0	18:0	18:1 ω-9	18:2 ω-6	18:3 ω-6	20:0	20:3 ω-6	20:4 ω-6	20:5 ω-3	22:6 ω-3	22:5 ω-3
**0***	24,11± 0,06	0,75 ± 0,06	50,73 ± 0,02	0,74 ± 0,02	0,91 ± 0,04	6,03 ± 0,10	0,71 ± 0,04	1,40 ± 0,02	1,01 ± 0,03	0,85 ± 0,03	0,87 ± 0,03	9,63 ± 0,03	1,36 ± 0,06	0,90 ± 0,18
**20***	21,36 ± 0,05	0,70 ± 0,01	48,57 ± 0,12	0,82 ± 0,08	0,82 ± 0,02	5,13 ± 0,05	0,81 ± 0,01	1,38 ± 0,03	1,07 ± 0,04	0,75 ± 0,03	0,62 ± 0,02	14,16 ± 0,02	3,03 ± 0,03	0,78 ± 0,03
**40***	18,56 ± 0,03	0,65 ± 0,04	43,93 ± 0,07	0,67 ± 0,05	0,65 ± 0,03	3,64 ± 0,06	0,71 ± 0,01	1,14 ± 0,01	0,86 ± 0,01	0,85 ± 0,03	0,94 ± 0,01	25,53 ± 0,02	0,93 ± 0,01	0,93 ± 0,02
**50***	20,04 ± 0,04	0,52 ± 0,02	47,16 ± 0,02	0,63 ± 0,04	0,66 ± 0,05	3,05 ± 0,05	0,60 ± 0,02	0,88 ± 0,02	0,78 ± 0,03	0,81 ± 0,01	0,70 ± 0,02	20,12 ± 0,03	2,99 ± 0,05	1,05 ± 0,03
**60***	12,08 ± 0,03	0,86 ± 0,03	23,17 ± 0,01	0,73 ± 0,02	0,92 ± 0,02	4,16 ± 0,02	0,88 ± 0,03	0,93 ± 0,02	0,82 ± 0,02	0,86 ± 0,04	0,80 ± 0,03	28,35 ± 0,04	24,49 ± 0,06	0,95 ± 0,03
**80***	23,16 ± 0,04	0,86 ± 0,05	56,30 ± 0,06	0,66 ± 0,03	0,94 ± 0,01	0,54 ± 0,05	0,65 ± 0,06	0,62 ± 0,01	0,90 ± 0,02	0,24 ± 0,05	0,41 ± 0,03	11,86 ± 0,02	2,39 ± 0,01	0,45 ± 0,02
**100***	19,33 ± 0,04	0,61 ± 0,03	49,20 ± 0,02	0,84 ± 0,04	0,70 ± 0,06	0,75 ± 0,04	0,73 ± 0,05	0,74 ± 0,05	0,78 ± 0,03	0,57 ± 0,02	0,62 ± 0,06	19,05 ± 0,06	5,42 ± 0,02	0,66 ± 0,04
**SC-CO_2**	8,11 ± 0,04	0,80 ± 0,08	20,82 ± 0,05	1,01 ± 0,08	1,18 ± 0,08	2,08 ± 0,01	1,00 ± 0,04	1,05 ± 0,05	1,15 ± 0,06	0,79 ± 0,05	1,04 ± 0,06	32,53 ± 0,03	27,51 ± 0,03	0,93 ± 0,04

[a]Values shown are mean ± SD ($n = 3$).

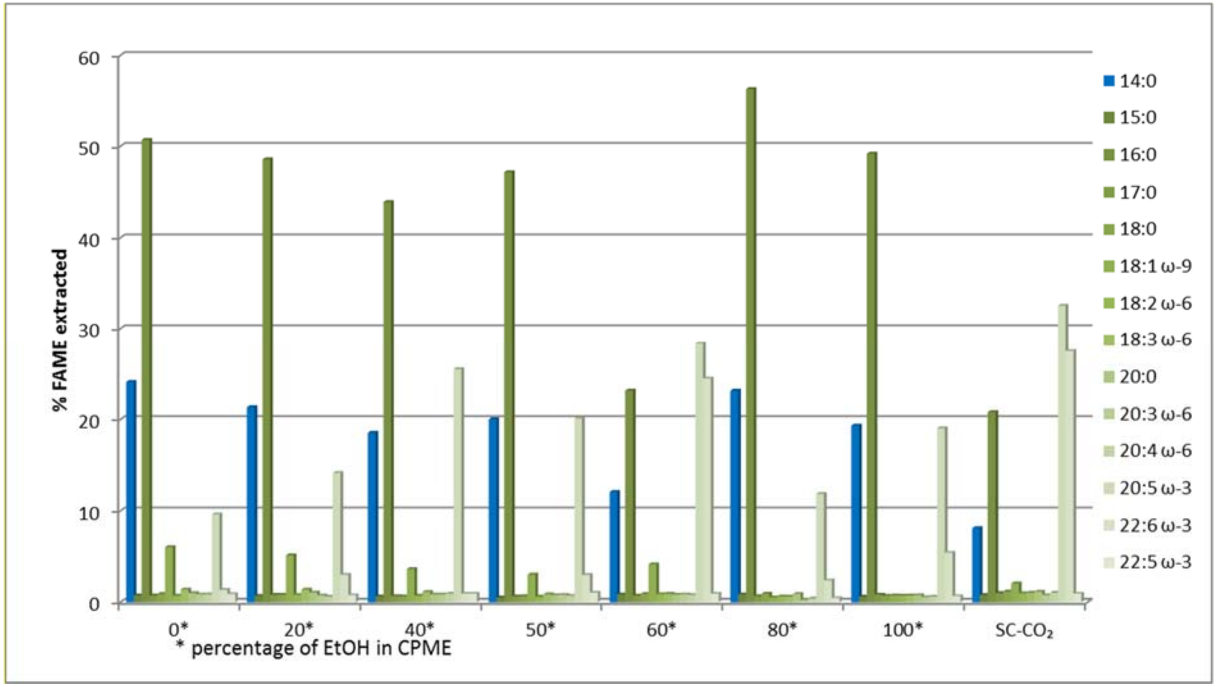

Figure 2. % Yield of all extracted FAMEs.

Method Validation LC-MS

The chromatogram was obtained scanning between 50 and 800 amu in negative ion mode (Figure, S3). From the information obtained in full scan mode and , it was possible to have information about the molecular weights of the fatty acids possibly present in the samples under investigation. From the molecular ions registered, the algal oil extract resulted to be composed of lauric acid (molecular ion at m/z 199.4); myristic acid (227.4); myristoleic acid (225.2); pentadecylic acid (241.4); palmitic acid (255.5); γ-linolenic acid (277.4); oleic acid (281.6); stearic acid (283.5); eicosapentanoic acid (301.6); docosaexaenoic acid (327.5); docosapentaenoic acid (329.7) and behenic acid (339.6). Successively, to attribute the molecular structures, on each single molecular ion previously recorded, experiments in product ion scan (MS/MS) were performed.

Table S2 lists the deprotonated molecules identified in full scan MS spectra, the fragment ions and the precursor ions identified by MS/MS experiments.

Table 2. List of the deprotonated molecules identified in full scan MS spectra of algal oil sample, fragment ions and precursor ions identified in MS/MS spectra.

ANALYTE	[M − H]-	PIS	PREC
**Lauric acid**	199.4	181.6; 155.0	399.7; 455.9
**Myristic acid**	227.4	209.5; 183.3	455.7; 483.8; 509.8
**Myristoleic acid**	225.2	207.2; 181.5	482
**Pentadecylic acid**	241.4	223.2; 197.2	483.8
**Palmitic acid**	255.5	237.6; 211.6	511.8; 537.9; 583.9
**γ-linolenic acid**	277.4	259.4; 233.4	555.8
**Oleic acid**	281.6	263.4; 237.4	509.8; 537.9; 563.8
**Stearic acid**	283.5	265.4; 239.2	540.5; 568
**Eicosapentanoic acid, EPA**	301.6	283.6; 257.6	602.2
**Docosahexaenoic acid, DHA**	327.5	283.6; 309.2; 229.6	583.9; 610.0; 656.0; 658.0
**Docosapentaenoic acid, DPA**	329.7	311.7; 285.6	658
**Behenic acid**	339.6	321.3; 294.9	596.5; 569.2

The initial complexity of the mass spectrum was therefore reduced when the product ion scan and a precursor ion scan were performed. ESI-MS/MS analysis were carried out for all the ions present in the full scan chromatogram for each algae extract. Ions included in the range of m/z 455-656 indicate molecular adducts between two identical or different fatty acid compounds (Table S3).

Table 3. Molecular composition of adduct with negative molecular ion between m/z 455 and m/z 656.

Negative ion	Fatty acid composition
**455.8**	2*myristic
**483.9**	2* Pentadecylic
**511.9**	2*palmitic
**555.8**	2*γ-linolenic
**563.8**	2*oleic
**583.9**	DHA+palmitic
**610.0**	DHA+oleic
**656.0**	2*DHA
**658.0**	DHA+DPA

For a better understanding of the type of interaction occurring between the fatty acids present into the algae extracts, particular standard solutions were prepared and analyzed. In particular, the solution under investigation were: a long chain polyunsaturated fatty acid (DHA), a long chain saturated fatty acid (stearic acid), a short chain fatty acid (butyric acid) and a solution containing all of them. The full scan spectrum of the DHA standard solution shows the deprotonated molecular ion at m/z 327.5 and ions at m/z 655.8 and 984.3, adducts of two and three DHA molecules, respectively. The stearic acid solution full scan spectrum

shows the deprotonated molecular ion at m/z 283.3, and ions at *m/z* 567.7 and 852.2, referring to two and three interacting acid molecules. The analysis of the solution containing the three fatty acids (DHA acid, stearic acid and butyric acid) leads to a spectrum in which there are the deprotonated ions of the three acids and the ions formed by the reaction between DHA and stearic acids (Figure S4). Butyric acid does not form any adduct with the other two.

To investigate the reaction site interested in the formation of the saturated and unsaturated fatty acid adducts, a mixture containing oleic acid and palmitic acid was analyzed. From the full scan spectrum of this solution, it was evident the formation of an acid-alcohol adduct: the ion at m/z 255.2 represents the deprotonated molecule of palmitic acid; the ion at m/z 268.0 represents the deprotonated molecule of oleic alcohol, while the formation of the adduct is confirmed by the ion at m/z 524.2 (Figure S5).

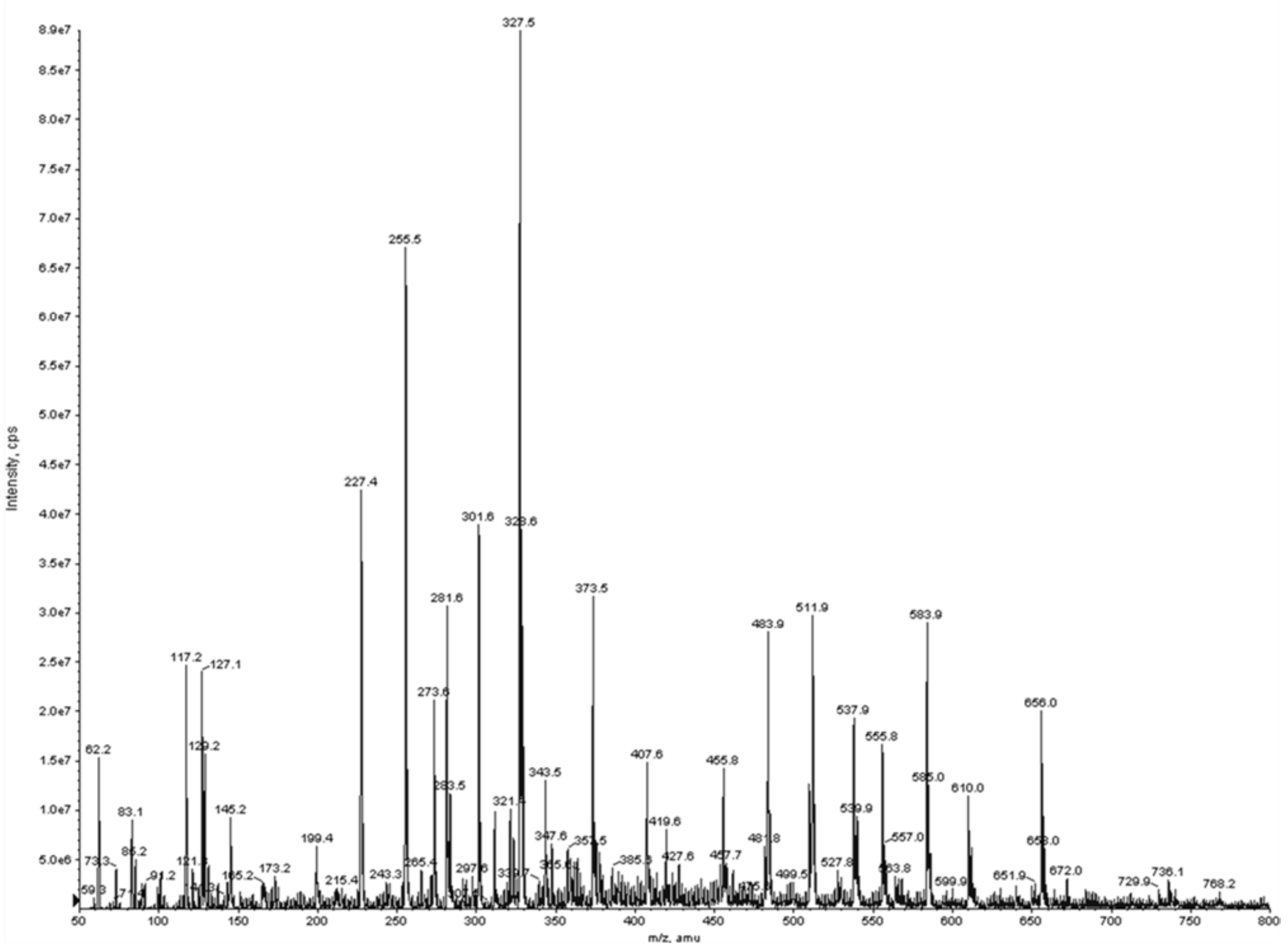

S3. Negative ESI full scan mass spectrum of algal oil sample.

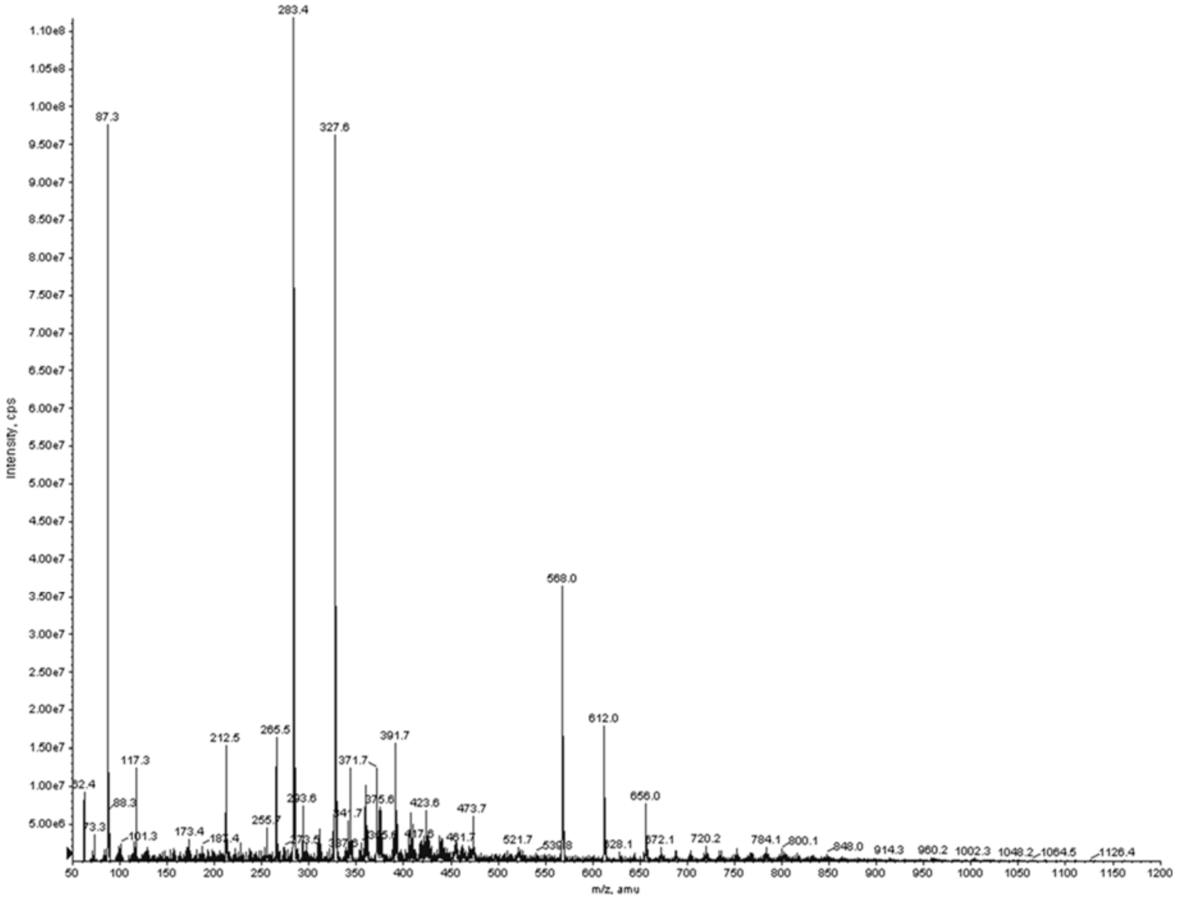

S4. Negative ESI full scan mass spectrum of standard solution of DHA, Stearic acid and butyric acid.

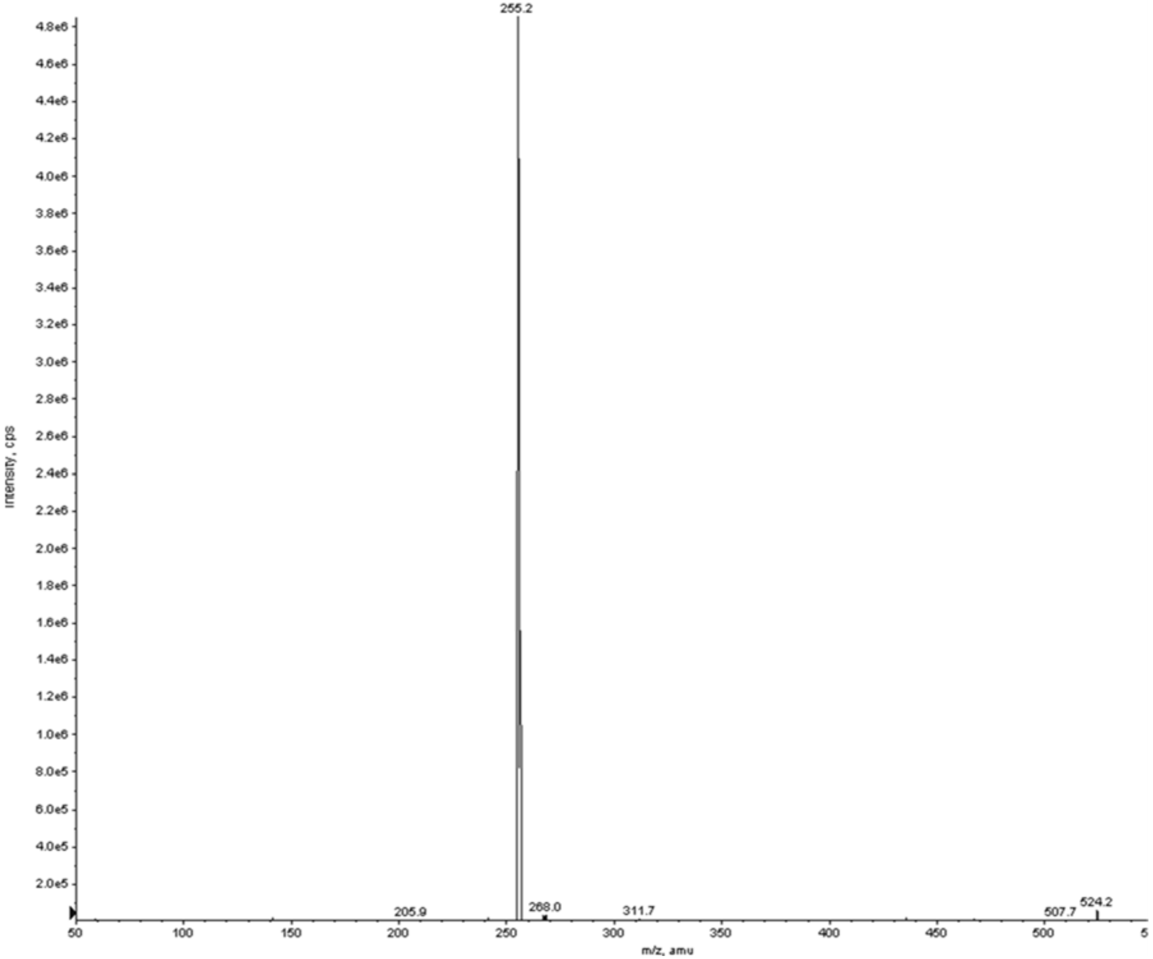

S5. Negative ESI full scan mass spectrum of standard solution of oleic alcohol and palmitic acid.
